# Neurocognitive Correlates of Diagnostic Heterogeneity in Children with ADHD: The Differential Contributions of Cognitive Disengagement Syndrome, Symptom Severity, and Anxiety

**DOI:** 10.3390/diagnostics16050808

**Published:** 2026-03-09

**Authors:** İbrahim Adak, Esin Özdeniz Varan, Nergis Eyüpoğlu, Ayşim Alpman, Zeynep Durmuş, Oğuz Bilal Karakuş, İpek Süzer Gamlı, Özalp Ekinci

**Affiliations:** 1 Department of Child and Adolescent Psychiatry, Erenkoy Mental and Neurological Diseases Training and Research Hospital, University of Health Sciences, 34736 Istanbul, Turkey; adakibrahim@hotmail.com (İ.A.); aysimalpmann@gmail.com (A.A.); zeynepbalta@windowslive.com (Z.D.); suzeripek@gmail.com (İ.S.G.); ozalpekinci@yahoo.com (Ö.E.); 2 Independent Researcher, 34736 Istanbul, Turkey; esinozdeniz@hotmail.com; 3 Department of Child and Adolescent Psychiatry, Trabzon Kanuni Training and Research Hospital, 61000 Trabzon, Turkey; bilal_k422@hotmail.com

**Keywords:** attention-deficit/hyperactivity disorder, cognitive disengagement syndrome, diagnostic assessment, memory, visuospatial function, anxiety, child

## Abstract

**Background/Objectives:** Attention-Deficit/Hyperactivity Disorder (ADHD) shows substantial cognitive heterogeneity, complicating individualized clinical formulation. This study examined whether Cognitive Disengagement Syndrome (CDS), anxiety, and ADHD symptom severity are associated with memory functions and visuospatial skills in children with ADHD. **Methods:** The sample included 120 children aged 6–12 years with ADHD (ADHD + CDS: *n* = 40; ADHD-only: *n* = 80). Memory was assessed with the Oktem Verbal Memory Processes Test (OVMPT) and Wechsler Memory Scale–Visual Reproduction (WMS–VR), and visuospatial skills with WISC-IV Block Design and Judgment of Line Orientation (JLO). ADHD symptoms were rated using combined parent–teacher Turgay-Diagnostic and Statistical Manual of Mental Disorders, Fourth Edition-Based Disruptive Behavior Disorders Scale (T-DSM-IV-S) scores; CDS symptoms with the Barkley Child Attention Scale; and anxiety with the SCARED-Child Form. Group comparisons, correlation analyses, and multivariable linear regression models were conducted. **Results:** The ADHD + CDS group performed worse on WISC-IV Block Design than the ADHD-only group (*p* = 0.005). In the ADHD + CDS group, inattention severity showed a strong negative association with WMS–VR short-term memory (*r* = −0.560, *p* < 0.001). In the ADHD-only group, inattention severity was negatively associated with OVMPT Spontaneous Recall (ρ = −0.319, *p* = 0.004) and JLO total score (ρ = −0.348, *p* = 0.002). Anxiety severity in the ADHD-only group was positively associated with OVMPT Total Learning (ρ = 0.350, *p* = 0.001), Highest Learning (ρ = 0.370, *p* = 0.001), and WMS–VR short-term memory (ρ = 0.304, *p* = 0.006). In regression analyses, the presence of CDS independently and negatively predicted WMS–VR short-term memory (β = −0.187, *p* = 0.018) and Block Design performances (β = −0.226, *p* = 0.016). Inattention symptom severity was also independently and negatively associated with Block Design performance (β = −0.243, *p* = 0.013). **Conclusions:** CDS status and symptom dimensions contribute to cognitive variability in pediatric ADHD, with CDS showing independent associations with timed visuospatial construction and short-term visual memory. Inattention severity emerged as a robust dimensional predictor of cognitive inefficiency across domains, supporting the clinical utility of symptom-based cognitive profiling in ADHD diagnostic evaluations. In addition, mild anxiety symptoms demonstrated meaningful associations with some learning and memory performances within the ADHD-only group, indicating that affective factors may modulate cognitive outcomes in ADHD. Taken together, these findings support considering CDS status and symptom dimensions jointly when characterizing cognitive variability in ADHD.

## 1. Introduction

Attention-deficit/hyperactivity disorder (ADHD) is a common neurodevelopmental disorder, affecting approximately 8% of the global pediatric population. Epidemiologically, ADHD is more common in boys than girls; recent global evidence suggests approximately 10% prevalence in boys versus 5% in girls [1]. It is characterized by persistent and developmentally inappropriate symptoms of inattention, hyperactivity and impulsivity that significantly impair functioning [2,3,4]. Beyond its core behavioral symptoms, ADHD is increasingly recognized as a diagnostically heterogeneous condition, with substantial variability in neurocognitive functioning across affected individuals [5,6]. This heterogeneity poses challenges for diagnostic formulation, as children meeting identical diagnostic criteria may exhibit markedly different cognitive profiles and functional needs.

Memory and visuospatial skills are among the cognitive domains most frequently implicated in ADHD, yet findings remain inconsistent. As a fundamental cognitive process supporting learning, development, and daily functioning, memory has been shown to display weaknesses across multiple domains [7]. While adult studies consistently demonstrate short-term verbal memory deficits [8,9], findings in children are mixed, with some reporting clear impairments and others suggesting largely preserved performance [10,11,12,13,14,15]. Visuospatial difficulties, which relate to the perception and organization of spatial information and enable effective interaction with the environment [16], have also been reported in ADHD, yet the profile of visuospatial skills appears heterogeneous across different tasks [17,18,19,20]. Executive functions may also contribute to this heterogeneity, as core processes such as working memory, inhibitory control, and processing speed can influence performance across both memory and visuospatial tasks [21,22].

From a diagnostic perspective, clarifying whether specific neurocognitive patterns map onto clinically relevant groups—such as ADHD with comorbid Cognitive Disengagement Syndrome (CDS)—is essential for improving diagnostic specificity. CDS, formerly termed sluggish cognitive tempo, is characterized by symptoms such as mental fogginess, hypoactivity, slowed processing, and fluctuating alertness [23]. Emerging evidence suggests that CDS may represent a distinct attentional phenotype within ADHD, raising the possibility that it is associated with a unique neurocognitive signature that could inform diagnostic decision-making [24,25,26].

Similarly, co-occurring anxiety symptoms frequently accompany ADHD and may influence cognitive test performance and executive-function estimates [27,28]. Understanding whether anxiety-related cognitive effects obscure or clarify the clinical interpretation of neuropsychological test results is crucial.

Given the cognitive heterogeneity of ADHD and inconsistent findings on memory and visuospatial functioning, we hypothesized that (1) children with ADHD and comorbid CDS would exhibit poorer memory and visuospatial performance than those without CDS, and (2) elevated anxiety symptoms and greater ADHD symptom severity would be related to poorer performance in these domains. Based on these hypotheses, we examined the effects of CDS, anxiety, and ADHD symptom severity on cognitive performance using standardized neuropsychological tests and structured clinical assessments.

## 2. Materials and Methods

This cross-sectional, single-center, clinic-based study examined memory and visuospatial performance in children with ADHD using group-based comparisons (ADHD with vs. without comorbid CDS), dimensional correlation analyses between symptom severity (anxiety and ADHD symptoms) and neuropsychological outcomes, multivariable linear regression analyses, and ROC curve analyses.

### 2.1. Participants

**Figure 1:**
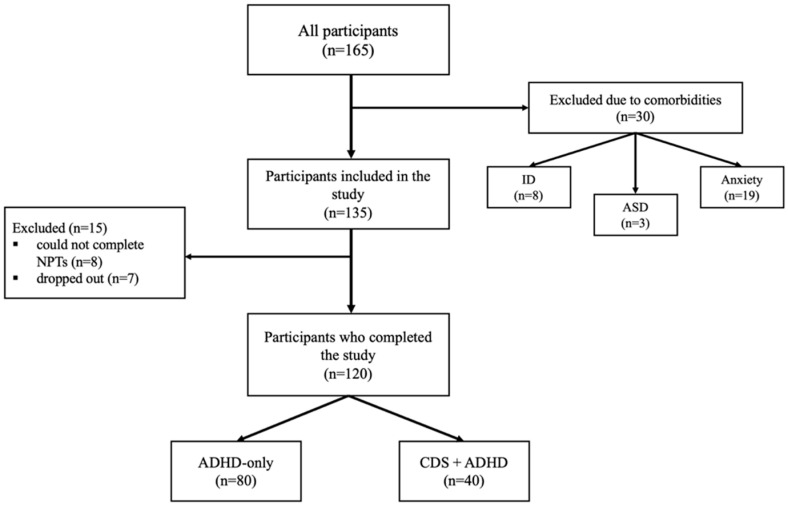
Participant selection diagram.

Participants were recruited from children presenting to the outpatient child and adolescent psychiatry clinic in Istanbul between January 2025 and June 2025. Inclusion criteria: Participants were eligible if they (1) were aged 6–12 years, (2) met Diagnostic and Statistical Manual of Mental Disorders, Fifth Edition, Text Revision (DSM-5-TR) criteria for ADHD, (3) had been receiving regular methylphenidate-based stimulant treatment for at least 2 months, and had not taken methylphenidate-based stimulants within the last 24 h prior to neuropsychological testing, (4) had no psychiatric comorbidity other than oppositional defiant disorder (ODD), (5) had no history of epilepsy or other chronic medical conditions that could affect memory functions, (6) had no visual or hearing impairment, and (7) did not have an intellectual disability. Exclusion criteria: Children were excluded if they (1) were younger than 6 years or older than 12 years, (2) met criteria for any psychiatric comorbidity other than ODD, (3) had epilepsy or other chronic medical conditions potentially affecting memory, (4) had visual or hearing impairment, (5) had an intellectual disability (defined as a Full-Scale IQ < 70 on the Wechsler Intelligence Scale for Children—Fourth Edition [WISC-IV]), or (6) did not complete the neuropsychological assessment or withdrew from the study. A total of 165 children with ADHD were initially enrolled; 30 were excluded due to psychiatric comorbidities, and 15 were excluded due to not completing the neuropsychological tests (*n* = 8) or withdrawing from the study (*n* = 7). The final sample consisted of 120 participants: 40 were diagnosed with ADHD and comorbid CDS, and 80 were diagnosed with ADHD only (see Figure 1).

### 2.2. Measures

All participants were evaluated by child and adolescent psychiatrists using structured clinical interviews, including the Kiddie Schedule for Affective Disorders and Schizophrenia—Present and Lifetime Version (K-SADS-PL), in accordance with DSM-5-TR criteria. Diagnostic evaluations were supported by review of available medical records.

#### 2.2.1. Assessment Procedure

All assessments were conducted in the child and adolescent psychiatry outpatient clinic. The diagnostic interview typically lasted 30–60 min. Parent-rated measures were completed by the primary caregiver (mother or father), child self-report measures were completed by the child, and teacher ratings were obtained from teachers. Neuropsychological tests were administered individually to children in a quiet room with the child alone in the testing room while the caregiver waited outside; the testing session took approximately 45–90 min in total, with short breaks provided as needed. All neuropsychological tests were administered and scored by the same experienced clinical psychologist according to standardized administration and scoring procedures based on the test manuals in order to ensure consistency. All participants had been receiving ADHD pharmacotherapy (methylphenidate-based stimulants) for at least 2 months, as documented in medical records and caregiver reports. Medication was withheld on the test day, with the last dose taken on the preceding day (approximately a 24 h washout), to minimize acute effects on neuropsychological test performance.

#### 2.2.2. The Kiddie Schedule for Affective Disorders and Schizophrenia—Present and Lifetime Version (K-SADS-PL)

The K-SADS-PL is a semi-structured diagnostic interview developed by Kaufman et al. [29] to assess both current and past psychiatric disorders in children and adolescents according to DSM criteria. The Turkish adaptation of the updated DSM-5 version of K-SADS-PL was conducted by Unal et al. [30]. The interviews were conducted by child and adolescent psychiatry physicians who had received structured training in the administration of the K-SADS-PL, and were certified in its use.

#### 2.2.3. Sociodemographic Information Form

This form was developed by the researchers to collect information from participants and their caregivers, including their sociodemographic information. The form covered a broad range of variables, including basic sociodemographic characteristics and family, developmental, and clinical background information. Although the form included multiple variables, only those directly relevant to the predefined study aims were included in the statistical analyses. Psychiatric family history was defined as the presence of any psychiatric disorder in first-degree relatives (mother, father, and/or siblings) based on caregiver reports.

#### 2.2.4. The Teacher and Parent Turgay Diagnostic and Statistical Manual of Mental Disorders, Fourth Edition-Based Child and Adolescent Disruptive Behavior Disorders Screening and Rating Scale (T-DSM-IV-S)

The T-DSM-IV-S is a 41-item instrument developed by Turgay according to DSM-IV criteria [31], and its Turkish validity and reliability were later established by Ercan and colleagues [32]. It assesses five symptom domains: inattention (9 items), hyperactivity (6 items), impulsivity (3 items), ODD (8 items), and conduct disorder (15 items). Items are rated on a four-point Likert scale, with higher scores reflecting greater symptom severity. In the present study, the scale was administered to both parents and teachers in order to evaluate ADHD symptom severity.

#### 2.2.5. Barkley Child Attention Scale (BCAS)

The BCAS is a 12-item parent-report instrument developed to measure CDS symptoms in children [33]. Each item is rated on a four-point scale, with higher scores indicating more pronounced CDS characteristics. The scale has been validated and shown to be reliable in the Turkish population [34]. In the present study, it was used to assess the presence of CDS symptoms.

#### 2.2.6. The Screen for Child Anxiety-Related Emotional Disorders (SCARED)—Child Form

The SCARED—Child form is a 41-item self-report scale developed by Birmaher et al. to screen for anxiety disorders in children [35]. Each item is rated on a three-point Likert scale with higher scores indicating more severe anxiety symptoms. The Turkish validity and reliability study of the child form was conducted by Karaceylan [36]. In the present study, the SCARED—Child Form was used to assess anxiety symptoms.

#### 2.2.7. Neuropsychological Tests

To evaluate visuospatial and memory functions, four neuropsychological tests were administered: the Oktem Verbal Memory Processes Test (OVMPT), the Visual Reproduction Subtest of the Wechsler Memory Scale (WMS–VR), the Block Design Subtest of the WISC-IV (WISC-IV Block Design), and the Judgment of Line Orientation Test (JLO).

##### Oktem Verbal Memory Processes Test (OVMPT)

The OVMPT was administered to assess multiple components of verbal learning and memory, including immediate recall, learning across trials, long-term storage, and retrieval. It is a standardized Turkish verbal memory test that includes three fixed 15-word forms (Lists A, B, and C); in the present study, List A was administered according to the test manual [37]. The test consists of a 15-word list that is read aloud to the participant at a pace of one word per second, and after each trial the participant is asked to freely recall as many words as possible, in any order. This procedure is repeated for up to ten trials. After a 30 min delay, participants were again asked to recall the words; if words were missing, a recognition list consisting of 15 targets and 30 distractors was administered. Following test administration, several scores were calculated. The learning-related scores included: Immediate Memory (words recalled after the first trial), Total Learning (sum across ten trials), Highest Learning (best performance in a single trial), False Learning (intrusions), and Perseverations (repeated intrusions). The long-term memory-related scores were evaluated after a 30 min delay and included: Spontaneous Recall (uncued recall), False Recall (intrusions at delay), Recognition (correctly identified targets), False Recognition (endorsed distractors), and Total Recall (sum of delayed recall and recognition). Pediatric normative data for Turkish children have been reported, supporting standardized use in pediatric samples [38].

##### Wechsler Memory Scale—Visual Reproduction (WMS–VR)

The WMS-VR subtest was administered to assess visual memory [39]. The test consists of three cards containing geometric figures that participants reproduce from memory by drawing. Following adaptations for the participants’ age, the second card was excluded, the two figures on the third card were presented separately for 10 s each, and a recognition format was added. These adjustments were made to reduce organizational demands that might confound visual memory performance in younger children and were applied uniformly to all participants. Each figure was scored on predetermined criteria. Based on these scores, short-term (immediate) and long-term (delayed, 30 min) memory scores were calculated, with higher scores indicating better memory performance.

##### Judgment of Line Orientation Test (JLO)

The JLO was used to assess spatial perception and orientation [40]. On each trial, participants were shown a pair of angled lines and asked to identify, among 11 reference lines, the two lines that had the same orientation as the target pair. Each correct response was scored as 1 and each incorrect response as 0, with higher total scores indicating better spatial perception and orientation.

##### Wechsler Intelligence Scale for Children—Fourth Edition, Block Design (WISC-IV Block Design)

The Block Design subtest assesses constructional ability [41]. The test materials consist of nine cubes (two sides entirely red, two sides entirely white, and two sides half red and half white) and a booklet containing 14 cards with printed designs. The participant is required to reproduce the designs shown on the cards within specified time limits by arranging the cubes with the appropriate faces. Scoring was based on item accuracy and, on later items, time-based bonus points, with higher scores reflecting better constructional ability. In the present study, raw scores were converted into age-adjusted scaled scores according to the WISC-IV manual, and these standardized scores were used for analyses.

### 2.3. Ethics

The study was approved by the institutional ethics committee of the hospital where the Child and Adolescent Psychiatry clinic is located. Written informed consent was obtained from parents/legal guardians before participation and prior to any study-related procedures.

### 2.4. Statistical Analysis

IBM SPSS (Statistical Package for the Social Sciences) Statistics 26.0 (IBM Corp., Armonk, NY, USA) was used for data analysis. The demographic and clinical characteristics of the participants were examined using descriptive statistics (number, percentage, mean, median, etc.). The distributional properties of continuous variables were assessed with the Shapiro–Wilk test. To evaluate between-group differences, sociodemographic and clinical variables were compared using independent samples t-tests and chi-square tests. Neuropsychological performance comparisons were conducted using t-tests and Mann–Whitney U tests, and effect sizes were reported as Cohen’s d and r. Associations between clinical symptom severity and neuropsychological performance were examined using Spearman correlations, and the Benjamini–Hochberg false discovery rate (FDR) correction was applied. In addition, multiple linear regression analyses were performed to identify predictors of WISC-IV Block Design and WMS-VR STM performance while controlling for covariates. ROC analyses were conducted using Python 3.13 (64-bit version). To evaluate group discriminability, the area under the curve (AUC), as an overall indicator of diagnostic accuracy, was calculated for the SCARED and WISC-IV Block Design scores. The optimal cutoff point was determined based on the Youden Index (sensitivity + specificity − 1). At the selected threshold, sensitivity, specificity, positive predictive value (PPV), and negative predictive value (NPV) were calculated. Because higher scores on the WISC-IV Block Design variable represent a more favorable clinical condition, the score direction was statistically adjusted prior to analysis to ensure correct interpretation of classification. Statistical tests were interpreted using a significance level of *p* < 0.05.

## 3. Results

### 3.1. Group Comparisons

The present study was completed by 120 participants diagnosed with ADHD. Among these, 40 participants had comorbid CDS (ADHD + CDS), while 80 did not (ADHD-only). Sociodemographic characteristics of the sample are shown in Table 1. The mean age of the entire sample was 9.66 years, and a predominance of male participants was observed. There was no significant difference between the ADHD + CDS and ADHD-only groups in terms of age and gender (*p* > 0.05). ODD was present in 2 children (5.0%) in the ADHD + CDS group and in 6 children (7.5%) in the ADHD-only group, with no significant between-group difference (*p* > 0.05).

Table 2 presents the comparison of neuropsychological test scores and clinical symptom severity scores between the ADHD + CDS and ADHD-only groups. When the ADHD + CDS and ADHD-only groups were compared, statistically significant differences were observed on the WISC-IV Block Design subtest, anxiety symptom severity (SCARED—Total), and BCAS scores. The WISC-IV Block Design scores were significantly lower in the ADHD + CDS group compared to the ADHD-only group (*Z* = −2.787, *p* = 0.005). The SCARED—Total scores were significantly higher in the ADHD + CDS group than in the ADHD-only group (*Z* = −2.314, *p* = 0.021). BCAS scores were also significantly higher in the ADHD + CDS group compared to the ADHD-only group (*Z* = −7.746, *p* < 0.001).

**Figure 2:**
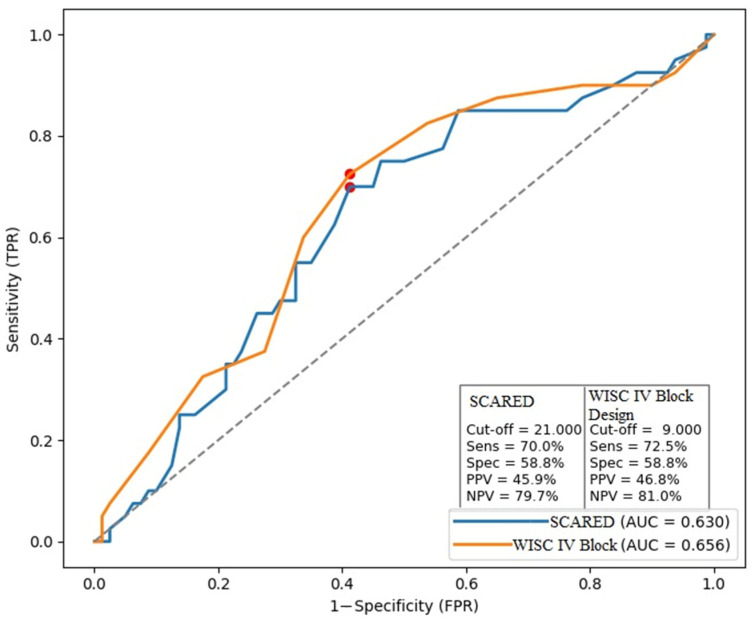
ROC analysis of the performance of SCARED—Total and WISC-IV Block Design scores in discriminating the ADHD + CDS group from the ADHD-only group. The red dots represent the optimal cut-off points obtained from ROC analysis for the SCARED total score and the WISC-IV Block Design score.

The effect size for WISC-IV Block Design was weak (*r* = 0.254), and the effect size for SCARED—Total was also weak (*r* = 0.211). Therefore, ROC analyses were conducted for WISC-IV Block Design and SCARED—Total scores, and the ROC curves are presented in Figure 2. For WISC-IV Block Design scores, the area under the curve (AUC) was 0.656. The optimal cutoff was 9.0, yielding a sensitivity of 72.5% and a specificity of 58.8%, with a positive predictive value (PPV) of 46.8% and a negative predictive value (NPV) of 81.0%. For SCARED—Total scores, the AUC was 0.630. The optimal cutoff was 21.0, yielding a sensitivity of 70.0% and a specificity of 58.8%, with a PPV of 45.9% and a NPV of 79.7% (Figure 2).

The effect size for BCAS scores was strong (*r* = 0.707). For all OVMPT and WMS–VR memory measures, effect sizes were weak (*r* = 0.001–0.139; WMS–VR *r* = 0.127). For clinical symptom scales (T-DSM-IV-S) with non-significant group differences, effect sizes were also weak (*r* = 0.028–0.096; *d* = 0.283). Although the difference in JLO total score was not statistically significant, the effect size was weak-to-moderate (*d* = 0.374).

### 3.2. Correlation Analysis

Table 3 presents the correlations between ADHD symptom scores (derived from combined parent and teacher T-DSM-IV-S ratings), anxiety scores (SCARED–total), and neuropsychological test performances in the ADHD + CDS and ADHD-only groups. After Benjamini–Hochberg false discovery rate (FDR) correction, a strong negative association was found in the ADHD + CDS group between Inattention Symptom Severity and WMS–VR STM performance (*r* = −0.560, *p* < 0.001, q = 0.036). Moderate negative associations were also observed between Inattention Symptom Severity and OVMPT—Spontaneous Recall (ρ = −0.319, *p* = 0.004, q = 0.036), as well as JLO Total Score (ρ = −0.348, *p* = 0.002, q = 0.024). In the ADHD-only group, after FDR correction, moderate positive associations were identified between Anxiety Symptom Severity and OVMPT—Total Learning (ρ = 0.350, *p* = 0.001, q = 0.018) and OVMPT—Highest Learning (ρ = 0.370, *p* = 0.001, q = 0.018). A moderate positive association was also found between Anxiety Symptom Severity and WMS–VR STM performance (ρ = 0.304, *p* = 0.006, q = 0.043).

Additionally, the correlations between ADHD symptom scores (derived from combined parent and teacher T-DSM-IV-S ratings), anxiety scores (SCARED–total), and neuropsychological test performances in the total sample are presented in Supplementary Table S1.

### 3.3. Regression Analyses

Table 4 shows that the multiple linear regression analysis yielded a model that significantly predicted the WISC-IV Block Design score, F(6,112) = 2.405, *p* = 0.032, R^2^ = 0.114 (adjusted R^2^ = 0.067). After controlling for age, sex, anxiety level, and inattention symptom severity, the presence of CDS was identified as an independent, negative and significant predictor of Block Design performance (B = −1.620, SE = 0.665, β = −0.226, *p* = 0.016, 95% CI [−2.937, −0.303]). Inattention symptom severity was also independently and negatively associated with WISC-IV Block Design performance (B = −0.118, SE = 0.047, β = −0.243, *p* = 0.013, 95% CI [−0.211, −0.026]).

Table 5 shows that the multiple linear regression analysis yielded a model that significantly predicted WMS-VR STM performance, F(6,112) = 11.648, *p* < 0.001, R^2^ = 0.384 (adjusted R^2^ = 0.351). Age was identified as a strong and positive predictor of WMS-VR STM performance (B = 0.946, SE = 0.149, β = 0.513, *p* < 0.001, 95% CI [0.651, 1.241]). The presence of CDS independently and negatively predicted WMS-VR STM performance after controlling for other variables (B = −1.192, SE = 0.495, β = −0.187, *p* = 0.018, 95% CI [−2.173,−0.211]).

## 4. Discussion

The present study aimed to examine whether memory and visuospatial measures provide clinically meaningful information for characterizing diagnostic heterogeneity in children with ADHD, with particular emphasis on the roles of CDS, symptom severity, and anxiety.

In our study, no significant sociodemographic differences were observed between the ADHD + CDS and ADHD-only groups. The groups were matched on mean age, and the proportion of males was higher in both groups, consistent with the general male predominance in ADHD [42]. Although the proportion of females was higher in the ADHD + CDS group (30% vs. 15%), this difference did not reach statistical significance, a pattern also reported by Krone et al. in adult samples [43]. Similarly, most studies have reported no reliable association between CDS and gender [23,44], although some found a relatively higher prevalence among girls [45,46].

From a diagnostic perspective, we examined whether memory and visuospatial measures capture a distinct neurocognitive profile associated with CDS within ADHD. When examining verbal memory with the OVMPT, contrary to our expectations, no significant differences emerged between the ADHD + CDS and ADHD-only groups. Consistent with the non-significant results, OVMPT effect sizes were weak, suggesting no practical group difference. Based on these findings, the presence of CDS does not appear to impose additional impairments on OVMPT performance. To our knowledge, no previous study has specifically compared ADHD and ADHD + CDS groups using the OVMPT. However, similar tasks such as the CVLT (California Verbal Learning Test) and the RAVLT (Rey Auditory Verbal Learning Test) have been used. In a pediatric sample including children with and without ADHD, Croghan found that CDS symptoms showed no significant correlation with RAVLT performance, although they were associated with slower processing speed [25]. In adults, Cerny et al. compared individuals with and without ADHD across high- and low-CDS subgroups and found no significant differences in CVLT performance [24]. Other studies have likewise failed to demonstrate consistent associations between CDS and verbal memory [47]. Overall, the literature aligns with our findings, suggesting that CDS is unlikely to directly affect verbal learning and memory.

Following verbal memory, we also assessed visual memory using the WMS–VR, which assesses both short-term and long-term visual memory. When the groups were compared in terms of WMS-VR performance using the Mann–Whitney U test, no statistically significant difference was observed, and the effect sizes were small. Therefore, a multiple linear regression analysis was conducted. The results indicated that age was a strong and positive predictor of short-term visual memory performance (B = 0.946, SE = 0.149, β = 0.513, *p* < 0.001, 95% CI [0.651, 1.241]), whereas the presence of CDS was a negative predictor (B = −1.192, SE = 0.495, β = −0.187, *p* = 0.018, 95% CI [−2.173, −0.211]). Although the Mann–Whitney U test did not reveal a statistically significant between-group difference in WMS-VR performance, the multivariable findings suggest a more nuanced pattern. In the multiple linear regression model, the strong positive predictor effect of age on visual memory performance may be related to normative developmental improvements in attentional control, encoding strategies, and memory organization across adolescence, whereas the negative predictive effect of CDS may reflect the sluggish alertness and reduced processing efficiency that interfere with effective encoding and retrieval processes. Taken together, these results imply that simple group comparisons may obscure CDS-related cognitive differences when substantial developmental variances are present, and they underscore the value of adjusting for key covariates when evaluating memory outcomes in heterogeneous ADHD samples. The literature directly addressing visual memory in ADHD + CDS is limited and shows mixed results. For example, Wu et al. reported poorer visual memory in ADHD + CDS children compared to ADHD-only peers [48], whereas Skirbekk found no association with absolute visual memory ability, but noted greater variability in spatial memory performance [47].

Studies assessing total memory (combining verbal and visual indices) have also reported controversial results. Baytunca et al. did not find significant differences in total memory scores between the ADHD + CDS and ADHD-only groups [45]. Similarly, İnci İzmir et al. found no additional impairment in memory measures, though group differences were observed in psychomotor speed [49]. In addition, while one study found no differences between CDS and control groups [50], another study suggested additive effects in total memory performance [51]. Based on the findings of the studies, it is difficult to directly interpret the effects of CDS on overall memory functions. Instead, CDS appears more reliably associated with attentional regulation, processing speed, and performance variability. Our findings on both OVMPT and WMS–VR indices are therefore in line with some of the literature, reinforcing the view that verbal memory may not be the primary cognitive domain affected by CDS, but short-term visual memory performance may be affected. Nevertheless, it should be kept in mind that these findings may partly reflect measurement constraints. Some memory indices (e.g., OVMPT) showed restricted score ranges (suggesting potential ceiling effects), and the age-adapted WMS–VR administration may have reduced sensitivity to subtle subgroup differences.

In the present study, children with ADHD + CDS obtained significantly lower scores than those with ADHD only on the WISC-IV Block Design subtest, suggesting additional difficulties in visuospatial construction, albeit with a weak effect size; clinically, this may present as slower and less efficient performance on time-limited visuospatial demands such as speeded copying, puzzle-like construction, and timed visual organization tasks and activities in the classroom. ROC analysis further indicated limited discriminative ability of Block Design for differentiating ADHD + CDS from ADHD-only (AUC = 0.656): although a cutoff score of 9 yielded relatively higher sensitivity (72.5%), specificity remained modest (58.8%) and the positive predictive value was low (46.8%), supporting that Block Design performance alone is insufficient as a diagnostic indicator of CDS within ADHD, while the higher negative predictive value (81.0%) suggests that relatively intact performance may modestly align with a lower likelihood of CDS in a broader clinical context. Importantly, multivariable regression refined this interpretation by showing that CDS presence remained an independent negative predictor of Block Design performance even after controlling for age, sex, anxiety level, and inattention symptom severity (β = −0.226, 95% CI [−2.937, −0.303]); inattention severity was also independently and negatively associated with performance (β = −0.243, 95% CI [−0.211, −0.026)), indicating that both CDS-related features and core attentional symptom burden contribute to visuospatial construction outcomes beyond demographic and clinical covariates. To date, research on visuospatial construction in ADHD with co-occurring CDS has been scarce and characterized by mixed results. While some studies linked higher CDS symptoms to weaker performance on tasks similar to Block Design [26], others using earlier WISC versions found no significant group differences [45]. The lower Block Design performance observed in our ADHD + CDS group may further be understood in light of two core features frequently emphasized in the CDS literature. First, slowed processing speed and vulnerability to time constraints are central characteristics of CDS [52]. Because Block Design is a timed subtest where both speed and accuracy contribute to the score, CDS may directly compromise performance. Second, difficulties in attentional regulation and alertness variability may also contribute. Completing Block Design requires sustained attention, consistent alertness, visuospatial processing, eye–hand coordination, and the ability to analyze and synthesize visual information into a coherent whole [53]. CDS, however, is often associated with mental fog, daydreaming, and inconsistent alertness, which can result in fluctuating performance on visuospatial tasks. Therefore, this finding should not be interpreted as reflecting visuospatial ability in isolation, and the potential contribution of time/speed components should also be considered. Accordingly, Block Design may be best taken as a supportive cognitive feature within comprehensive assessment rather than a standalone marker of CDS.

In addition, we found no significant differences between the ADHD + CDS and ADHD-only groups on the JLO, suggesting that basic visuospatial perception and orientation are relatively preserved. The present findings contribute to the growing literature on neurocognitive heterogeneity in ADHD by demonstrating that CDS is selectively associated with visuospatial construction difficulties, rather than global memory impairments. Importantly, this pattern suggests that not all cognitive weaknesses observed in ADHD are diagnostically informative. While OVMPT memory measures failed to distinguish ADHD subgroups, performance on a timed visuospatial construction task (Block Design) showed potential diagnostic relevance. Replication in larger samples will be important to determine whether the observed visuospatial construction weaknesses represent a core feature of CDS or reflect secondary effects of processing speed and attentional variability.

We also examined associations between cognitive performance and broader clinical characteristics, including ADHD symptom dimensions and anxiety. We examined these correlations separately within the ADHD + CDS and ADHD-only groups to explore potential subgroup-specific patterns. Our findings demonstrated that in the ADHD + CDS group, inattention symptom severity was strongly and negatively associated with WMS–VR short-term memory performance (*r* = −0.560, *p* < 0.001, q = 0.036), indicating that greater inattentive symptom burden was linked to poorer immediate visual memory/retention in this subgroup. In the ADHD-only group, inattention symptom severity showed moderate negative correlations with OVMPT Spontaneous Recall (ρ = −0.319, *p* = 0.004, q = 0.036) and JLO total score (ρ = −0.348, *p* = 0.002, q = 0.024), suggesting that higher inattention was related to reduced retrieval efficiency on verbal memory tasks and weaker visuospatial organization/orientation abilities. These findings suggest that inattentive symptoms may interfere with encoding and retrieval processes, resulting in less efficient learning and consolidation of information. Similar relationships between inattention symptom severity and memory weaknesses have been described in earlier research [54,55]. Some studies, however, did not replicate these associations [13,56], likely due to methodological and sample-related differences. In terms of visuospatial skills, inattention symptom severity was related to poorer performance in visuospatial perception and orientation. This finding is consistent with previous research demonstrating associations between inattention and visuospatial skill deficits in children [57]. While the correlational nature of this study precludes causal interpretation, the observed association may indicate that inattention contributes to difficulties in sustaining focus on visual–spatial details during perceptual tasks in children with ADHD.

The association between greater inattention symptom severity and poorer memory and visuospatial performance is noteworthy, as it suggests that increasing attentional difficulties may extend their impact to broader cognitive domains. Taken together, the observed association between inattention severity and poorer cognitive performance across domains further highlights the importance of dimensional symptom assessment. From a diagnostic standpoint, these findings support models that emphasize symptom severity and cognitive phenotype over categorical subtype classification alone.

Interestingly, anxiety symptoms demonstrated modest positive associations with some memory measures, including total and highest learning on verbal memory tasks, as well as short-term visual memory in the ADHD-only group. These findings should be interpreted cautiously and considered exploratory given the correlational design and modest effect sizes. While anxiety is often theorized to impair cognition by taxing attentional resources [27], recent findings suggest that co-occurring anxiety may, under some circumstances, be associated with relatively better performance on specific executive processes in youth with ADHD [28]. A possible explanation is that mild anxiety may be related to heightened vigilance or more cautious information processing, which could support performance on structured memory tasks. In this way, anxious arousal might reduce impulsive responding during testing, albeit at the cost of slower and more effortful processing [58,59]. Accordingly, the positive associations should not be interpreted as enhanced memory capacity, but rather as differences in performance efficiency during testing. Additionally, because anxiety levels in our sample were mild/subclinical, this pattern may be sample-specific and should be generalized cautiously, particularly to clinical anxiety disorders. Taken together, our findings highlight an important nuance: children with ADHD who have mild anxiety—but not at a pathological level—may show relatively preserved memory performance, rather than uniformly worse outcomes. Clinically, this pattern underscores the need for clinicians to contextualize test performance within emotional functioning when interpreting neuropsychological results.

The present study provides several clinically relevant insights for ADHD diagnostic practice. First, the selective impairment observed in visuospatial construction among children with ADHD and comorbid CDS suggests that tasks such as the WISC-IV Block Design may help to provide diagnostically relevant information when CDS is suspected. Unlike memory measures, which showed limited discriminatory value, visuospatial construction performance differentiated clinically meaningful subgroups. Clinically, CDS may be associated with difficulty in time-limited tasks with high constructional demands, which may guide individualized educational support targeting the areas of impairment.

Second, the robust associations between inattention symptom severity and neurocognitive performance emphasize the importance of dimensional symptom evaluation in diagnostic assessments. Elevated inattention scores may signal increased risk for broader cognitive inefficiencies, warranting more comprehensive neuropsychological evaluation even in the absence of additional diagnoses.

Finally, the nuanced role of anxiety highlights the need for integrative diagnostic interpretation. Anxiety-related differences in test engagement or response style may influence performance on structured tasks. Clinicians should therefore interpret neuropsychological findings within the broader emotional and behavioral context of the child.

A key strength of this study is the comprehensive assessment of memory and visuospatial skills through multiple standardized, performance-based tests administered by an experienced clinical psychologist. In addition, evaluations were not based solely on rating scales, as all participants underwent structured clinical interviews and medical-record review, providing multi-source clinical validation that likely reduced—though did not eliminate—informant-related bias. Collectively, our findings support the use of targeted neurocognitive testing as an adjunct to clinical diagnosis, enhancing diagnostic precision and individualized treatment planning in ADHD.

Our study has several limitations that should be considered when interpreting the findings. Its cross-sectional, single-center design conducted at a child and adolescent psychiatry outpatient clinic, together with the relatively modest sample size, may limit the generalizability of the results to other regions and healthcare settings. We also lacked a typically developing comparison group, preventing comparison with normative performance. In addition, excluding intellectual disability and most psychiatric comorbidities may limit generalizability to typical clinical ADHD samples. Reliance on self- and parent/teacher-report measures may have introduced some subjectivity or minor reporting bias. In particular, symptom measures relied on different informants and were also included in correlational analyses: CDS symptoms were measured via parent report (BCAS), whereas anxiety symptoms were assessed via child self-report (SCARED). Because parents’ perceptions of CDS-related behaviors may differ from the child’s subjective internalizing experiences, associations involving CDS and anxiety symptoms should be interpreted cautiously. While the adapted WMS–VR protocol was applied uniformly across participants to ensure internal comparability, the modifications may limit direct comparability with standard administration and normative references. A further limitation is that tests were administered in a fixed order for all participants; although short breaks were allowed when needed, order-related practice or fatigue effects cannot be fully ruled out. All participants were treated with methylphenidate-based stimulants and were assessed after a ≈24 h washout to reduce acute effects; nevertheless, short-term withdrawal effects cannot be ruled out, and long-term treatment may exert persistent cognitive influences beyond the washout period. Because we did not include a comprehensive executive-function battery, we could not directly test the extent to which executive demands account for variability in memory and visuospatial outcomes. In addition, we did not include a direct processing-speed measure, which limits interpretation. Future studies should replicate these findings in larger, multi-center and longitudinal samples and include comprehensive executive-function measures to clarify underlying mechanisms.

## 5. Conclusions

Overall, comorbid CDS in children with ADHD was most consistently associated with poorer performance on a timed visuospatial construction measure (WISC-IV Block Design), while visual memory measures showed limited but suggestive differentiation and verbal memory measures did not reliably distinguish ADHD + CDS from ADHD only at the group level. Dimensional inattention emerged as a clinically informative correlate of cognitive inefficiency across certain domains. In addition, anxiety symptoms demonstrated meaningful associations with some learning and memory performances within the ADHD-only group, indicating that affective factors may modulate cognitive outcomes in ADHD. Taken together, these findings support considering CDS status and symptom dimensions jointly when characterizing cognitive variability in ADHD.

## Figures and Tables

**Table 1:** Sociodemographic Characteristics of the Study Sample.

Variables	ADHD + CDS (*n* = 40)	ADHD-Only (*n* = 80)	Statistical Analysis	*p* Value
Age, med (IQR)	9.66 (8.09–11.25)	9.75 (8.33–11.00)	*Z* = 0.759	0.306
Gender (Male/Female)	28/12	68/12	*χ*^2^ = 3.750	0.090
Maternal age, med (IQR)	39.50 (34.50–45.00)	39.00 (34.00–44.00)	*Z* = −0.061	0.951
Paternal age, M (SD)	42.75 (6.08)	42.85 (6.18)	*t* = 0.084	0.933
Presence of psychiatric family history, *n* (%)	12 (30.0)	21 (26.2)	*χ*^2^ = 0.047	0.828
Comorbid ODD, *n* (%)	2 (5.0)	6 (7.5)	*χ*^2^ = 0.268	0.717

Note. ADHD = Attention-Deficit/Hyperactivity Disorder; CDS = Cognitive Disengagement Syndrome; ODD: oppositional defiant disorder, *t* = independent samples *t*-test; *χ*^2^ = chi-square test; *M* = mean; *SD* = standard deviation; *med* = Median; *IQR* = interquartile range (1st and 3rd quartiles presented); *Z* = Mann–Whitney U test; *p* < 0.05 indicates statistical significance.

**Table 2:** Comparison of Neuropsychological Test Scores and Clinical Symptom Severity Scores Between ADHD + CDS and ADHD-only Groups.

Variables	ADHD + CDS (*n* = 40)	ADHD-Only (*n* = 80)	Statistical Analysis	*p* Value	Effect Size
OVMPT—Immediate Memory, med (IQR)	5.00 (5.00–6.00)	5.00 (4.00–6.00)	*Z* = −0.237	0.813	*r* = 0.022
OVMPT—Total Learning, med (IQR)	102.50 (89.00–114.75)	105.00 (91.00–119.00)	*Z* = 0.707	0.479	*r* = 0.065
OVMPT—Highest Learning, med (IQR)	13.00 (12.00–14.50)	14.00 (12.00–15.00)	*Z* = −1.517	0.129	*r* = 0.139
OVMPT—False Learning, med (IQR)	1.00 (0.00–1.00)	1.00 (0.00–2.00)	*Z* = 1.332	0.183	*r =* 0.121
OVMPT—Perseveration, med (IQR)	0.00 (0.00–0.00)	0.00 (0.00–0.00)	*Z* = 0.933	0.351	*r =* 0.085
OVMPT—Spontaneous Recall, med (IQR)	12.00 (10.00–14.00)	12.00 (10.25–13.00)	*Z* = 0.365	0.715	*r =* 0.034
OVMPT—Total Recall, med (IQR)	15.00 (15.00–15.00)	15.00 (15.00–15.00)	*Z* = 1.482	0.138	*r =* 0.135
OVMPT—False Recall, med (IQR)	0.00 (0.00–0.00)	0.00 (0.00–0.00)	*Z* = 0.008	0.994	*r =* 0.001
OVMPT—False Recognition, med (IQR)	0.00 (0.00–0.00)	0.00 (0.00–0.00)	*Z* = 0.338	0.735	*r =* 0.031
WMS–VR LTM, med (IQR)	7.00 (4.00–10.00)	8.00 (7.00–11.00)	*Z* = −1.388	0.165	*r* = 0.127
WMS–VR STM, med (IQR)	8.00 (5.00–11.00)	8.50 (7.00–11.00)	*Z* = −1.388	0.165	*r* = 0.127
WISC-IV Block Design, med (IQR)	8.00 (6.00–10.00)	10.00 (7.00–12.00)	*Z* = −2.787	**0.005**	*r* = 0.254
JLO—Total Score, M (SD)	16.15 (6.73)	18.49 (5.99)	*t =* −1.933	0.056	*d* = *0*.374
T-DSM-IV-S—Inattention Symptom Severity, med (IQR)	15.00 (12.50–19.50)	15.00 (8.50–21.00)	*Z* = −0.301	0.763	*r* = 0.028
T-DSM-IV-S—Hyperactivity/Impulsivity Symptom Severity, med (IQR)	9.50 (1.00–16.50)	10.00 (4.00–19.00)	*Z = *−1.050	0.294	*r* = 0.096
T-DSM-IV-S Toplam, M (SD)	33.18 (18.54)	39.04 (21.73)	*t* = 1.460	0.147	*d* = 0.283
SCARED—Total, med (IQR)	25.00 (18.00–35.50)	17.50 (12.00–29.00)	*Z* = −2.314	**0.021**	*r* = 0.211
BCAS, med (IQR)	26.00 (22.50–29.00)	16.00 (13.50–19.00)	*Z* = −7.746	**<0.001**	*r* = 0.707

Note. CDS = Cognitive Disengagement Syndrome; OVMPT = Oktem Verbal Memory Processes Test; WMS–VR = Wechsler Memory Scale—Visual Reproduction; STM = Short-term memory; LTM = Long-term memory; WISC-IV Block Design = Wechsler Intelligence Scale for Children—Fourth Edition, Block Design; JLO = Judgment of Line Orientation. SCARED = Screen for Child Anxiety-Related Emotional Disorders. BCAS = Barkley Child Attention Scale. *M* = Mean; *SD* = Standard deviation; *med* = Median; *IQR* = Interquartile range (1st and 3rd quartiles presented); *t* = independent samples t-test; *Z* = Mann–Whitney U test; *d* = Cohen’s d; *r* = Rank biserial correlation. Bold values indicate significant coefficients.

**Table 3:** Correlations between symptom severity (inattention, hyperactivity/impulsivity, and anxiety) and neuropsychological test performance in the ADHD + CDS and ADHD-only groups (ρ/*r*; raw *p* and BH–FDR corrected q).

	ADHD + CDS (*n* = 40)			ADHD-Only (*n* = 80)		
Neuropsychological Tests	Inattention Symptom Severity *r*/rho (*p*; q)	Hyperactivity/Impulsivity Symptom Severity *r*/rho (*p*; q)	Anxiety Symptom Severity *r*/rho (*p*; q)	Inattention Symptom Severity *r*/rho (*p*; q)	Hyperactivity/Impulsivity Symptom Severity *r*/rho (*p*; q)	Anxiety Symptom Severity *r*/rho (*p*; q)
OVMPT—Immediate Memory	−0.405 ^b^ (*p* = 0.010; q = 0.093)	−0.189 ^b^ (*p* = 0.242; q = 0.502)	0.080 ^b^ (*p* = 0.624; q = 0.732)	−0.219 ^b^ (*p* = 0.051; q = 0.223)	0.057 ^b^ (*p* = 0.613; q = 0.773)	0.198 ^b^ (*p* = 0.078; q = 0.255)
OVMPT—Total Learning	−0.379 ^a^ (*p* = 0.016; q = 0.093)	−0.258 ^b^ (*p* = 0.108; q = 0.299)	0.339 ^a^ (*p* = 0.032; q = 0.144)	−0.137 ^b^ (*p* = 0.225; q = 0.450)	0.063 ^b^ (*p* = 0.580; q = 0.773)	**0.350 ^b^ (** ***p*** ** = 0.001; q = 0.018)**
OVMPT—Highest Learning	−0.110 ^b^ (*p* = 0.498; q = 0.690)	−0.205 ^b^ (*p* = 0.205; q = 0.461)	0.300 ^b^ (*p* = 0.060; q = 0.240)	−0.175 ^b^ (*p* = 0.121; q = 0.290)	0.036 ^b^ (*p* = 0.749; q = 0.843)	**0.370 ^b^ (** ***p*** ** = 0.001; q = 0.018)**
OVMPT—False Learning	0.278 ^b^ (*p* = 0.083; q = 0.264)	0.247 ^b^ (*p* = 0.124; q = 0.319)	0.144 ^b^ (*p* = 0.375; q = 0.609)	0.222 ^b^ (*p* = 0.047; q = 0.223)	−0.101 ^b^ (*p* = 0.375; q = 0.587)	−0.147 ^b^ (*p* = 0.193; q = 0.434)
OVMPT—Perseveration	0.165 ^b^ (*p* = 0.310; q = 0.531)	0.110 ^b^ (*p* = 0.498; q = 0.690)	−0.094 ^b^ (*p* = 0.565; q = 0.701)	0.009 ^b^ (*p* = 0.937; q = 0.937)	−0.048 ^b^ (*p* = 0.671; q = 0.805)	−0.181 ^b^ (*p* = 0.108; q = 0.290)
OVMPT—Spontaneous Recall	−0.102 ^b^ (*p* = 0.532; q = 0.690)	−0.007 ^b^ (*p* = 0.965; q = 0.965)	0.373 ^b^ (*p* = 0.018; q = 0.093)	**−0.319 ^b^ (** ***p*** ** = 0.004; q = 0.036)**	−0.177 ^b^ (*p* = 0.117; q = 0.290)	0.209 ^b^ (*p* = 0.062; q = 0.223)
OVMPT—False Recall	0.170 ^b^ (*p* = 0.294; q = 0.529)	−0.130 ^b^ (*p* = 0.425; q = 0.637)	−0.290 ^b^ (*p* = 0.070; q = 0.252)	0.141 ^b^ (*p* = 0.213; q = 0.450)	−0.025 ^b^ (*p* = 0.827; q = 0.876)	−0.019 ^b^ (*p* = 0.869; q = 0.894)
OVMPT—Total Recall	−0.186 ^b^ (*p* = 0.251; q = 0.502)	0.079 ^b^ (*p* = 0.630; q = 0.732)	−0.223 ^b^ (*p* = 0.167; q = 0.401)	0.056 ^b^ (*p* = 0.623; q = 0.773)	0.117 ^b^ (*p* = 0.303; q = 0.496)	0.210 ^b^ (*p* = 0.061; q = 0.223)
OVMPT—False Recognition	0.022 ^b^ (*p* = 0.892; q = 0.944)	−0.053 ^b^ (*p* = 0.744; q = 0.812)	0.273 ^b^ (*p* = 0.088; q = 0.264)	−0.081 ^b^ (*p* = 0.473; q = 0.681)	−0.123 ^b^ (*p* = 0.275; q = 0.489)	−0.121 ^b^ (*p* = 0.285; q = 0.489)
WMS–VR STM	**−0.560 ^a^ (** ***p*** ** < 0.001; q = 0.036)**	−0.382 ^b^ (*p* = 0.015; q = 0.093)	0.140 ^a^ (*p* = 0.389; q = 0.609)	−0.181 ^b^ (*p* = 0.108; q = 0.290)	−0.029 ^b^ (*p* = 0.798; q = 0.871)	**0.304 ^b^ (** ***p*** **= 0.006; q = 0.043)**
WISC-IV Block Design	−0.070 ^b^ (*p* = 0.669; q = 0.753)	−0.008 ^b^ (*p* = 0.962; q = 0.965)	−0.175 ^b^ (*p* = 0.281; q = 0.529)	−0.252 ^b^ (*p* = 0.024; q = 0.144)	0.083 ^b^ (*p* = 0.464; q = 0.681)	0.041 ^b^ (*p* = 0.717; q = 0.833)
JLO—Total Score	−0.380 ^a^ (*p* = 0.016; q = 0.093)	−0.411 ^b^ (*p* = 0.008; q = 0.093)	−0.100 ^a^ (*p* = 0.537; q = 0.690)	**−0.348 ^b^ (** ***p*** ** = 0.002; q = 0.024)**	−0.122 ^b^ (*p* = 0.281; q = 0.489)	0.068 ^b^ (*p* = 0.550; q = 0.762)

Note. OVMPT = Oktem Verbal Memory Processes Test; WMS–VR STM = Wechsler Memory Scale–Visual Reproduction Short-Term Memory; WISC–IV Block Design = Wechsler Intelligence Scale for Children—Fourth Edition, Block Design; JLO = Judgment of Line Orientation. Symptom severity scores for Inattention and Hyperactivity/Impulsivity were derived from the T-DSM-IV-S and anxiety symptom severity scores were obtained from the total score of the SCARED. Values are presented as correlation coefficients, with the raw *p* value and the Benjamini–Hochberg FDR-corrected *p* value (q) provided in parentheses. ^a^ = Pearson correlation coefficient (*r*), ^b^ = Spearman rank correlation coefficient (ρ). FDR correction was applied separately within each group (36 comparisons per group). Bold values indicate associations that remained significant at q < 0.05. Correlation strength was interpreted as strong (|*r*| ≥ 0.50), moderate (0.30 ≤ |*r*| < 0.50), and weak (|*r*| < 0.30).

**Table 4:** Multiple Linear Regression Analysis of Independent Predictors of WISC-IV Block Design Performance.

	Unstandardized Coefficients		Standardized Coefficients	*t*	*p*	95.0% CI
	B	SE	β			
(Constant)	13.097	2.348		5.578	<0.001	8.445–17.748
Age	−0.097	0.200	−0.047	−0.486	0.628	−0.494–0.299
Gender (Girl)	−0.541	0.812	−0.063	−0.666	0.507	−2.151–1.068
Group (ADHD + CDS)	−1.620	0.665	−0.226	−2.437	**0.016**	−2.937–−0.303
Anxiety (SCARED—Total)	0.011	0.025	0.042	0.447	0.656	−0.038–0.060
Inattention Symptom Severity	−0.118	0.047	−0.243	−2.529	**0.013**	−0.211–−0.026

Note. Symptom severity scores for Inattention were derived from the T-DSM-IV-S. Unstandardized coefficients (B), standard errors (SE), standardized coefficients (β), *t* values, *p* values, and 95% confidence intervals (CI) are presented in the table. Model statistics were F(6,112) = 2.405, *p* = 0.032; R^2^ = 0.114; adjusted R^2^ = 0.067. No multicollinearity was detected (Tolerance > 0.20 and VIF < 5 for all variables). Statistical significance was set at *p* < 0.05. Bold values indicate statistically significant results (*p* < 0.05).

**Table 5:** Multiple Linear Regression Analysis of Independent Predictors of WMS-VR Short-Term Memory Performance.

	Unstandardized Coefficients		Standardized Coefficients	*t*	*p*	95.0% CI
	B	SE	β			
(Constant)	0.647	1.749		0.370	0.712	−2.818–4.112
Age	0.946	0.149	0.513	6.346	**<0.001**	0.651–1.241
Gender (Girl)	0.896	0.605	0.117	1.482	0.141	−0.302–2.095
Group (ADHD + CDS)	−1.192	0.495	−0.187	−2.407	0.018	−2.173–−0.211
Anxiety (SCARED—Total)	0.029	0.018	0.124	1.591	0.114	−0.007–0.066
Inattention Symptom Severity	−0.047	0.035	−0.108	−1.349	0.180	−0.116–0.022

Note. Symptom severity scores for Inattention were derived from the T-DSM-IV-S. Unstandardized coefficients (B), standard errors (SE), standardized coefficients (β), *t* values, *p* values, and 95% confidence intervals (CI) are presented in the table. Model statistics were F(6,112) = 11.648, *p* < 0.001; R^2^ = 0.384; adjusted R^2^ = 0.351. No multicollinearity was detected (Tolerance > 0.20 and VIF < 5 for all variables). Statistical significance was set at *p* < 0.05. Bold values indicate statistically significant results (*p* < 0.05).

## Data Availability

Due to ethical and legal restrictions related to patient confidentiality, the minimal dataset cannot be shared publicly. The data are available from the corresponding author upon reasonable request.

## Supplementary Materials

The following supporting information can be downloaded at: https://www.mdpi.com/article/10.3390/diagnostics16050808/s1, Table S1: Correlations Between ADHD Symptom Severity, Anxiety Severity, and Neuropsychological Test Performances (*n* = 120).

## Abbreviations

ADHD	Attention-Deficit/Hyperactivity Disorder
CDS	Cognitive Disengagement Syndrome
DSM-5-TR	Diagnostic and Statistical Manual of Mental Disorders, Fifth Edition, Text Revision
K-SADS-PL	Kiddie Schedule for Affective Disorders and Schizophrenia—Present and Lifetime Version
T-DSM-IV-S	Turgay Diagnostic and Statistical Manual of Mental Disorders, Fourth Edition-Based Disruptive Behavior Disorders Scale
ODD	Oppositional defiant disorder
BCAS	Barkley Child Attention Scale
SCARED	Screen for Child Anxiety Related Emotional Disorders
OVMPT	Oktem Verbal Memory Processes Test
WMS–VR	Wechsler Memory Scale—Visual Reproduction
WISC-IV	Wechsler Intelligence Scale for Children—Fourth Edition
JLO	Judgment of Line Orientation
SPSS	Statistical Package for the Social Sciences
STM	Short-Term Memory
LTM	Long-Term Memory

## References

[1] Ayano G., Demelash S., Gizachew Y., Tsegay L., Alati R. (2023). The Global Prevalence of Attention Deficit Hyperactivity Disorder in Children and Adolescents: An Umbrella Review of Meta-Analyses. J. Affect. Disord..

[2] American Psychiatric Association Publishing (2022). American Psychiatric Association Diagnostic and Statistical Manual of Mental Disorders; DSM-5-TR.

[3] Arnold L.E., Hodgkins P., Kahle J., Madhoo M., Kewley G. (2020). Long-Term Outcomes of ADHD: Academic Achievement and Performance. J. Atten. Disord..

[4] French B., Nalbant G., Wright H., Sayal K., Daley D., Groom M.J., Cassidy S., Hall C.L. (2024). The Impacts Associated with Having ADHD: An Umbrella Review. Front. Psychiatry.

[5] Faraone S.V., Bellgrove M.A., Brikell I., Cortese S., Hartman C.A., Hollis C., Newcorn J.H., Philipsen A., Polanczyk G.V., Rubia K. (2024). Attention-Deficit/Hyperactivity Disorder. Nat. Rev. Dis. Primer.

[6] Luo Y., Weibman D., Halperin J.M., Li X. (2019). A Review of Heterogeneity in Attention Deficit/Hyperactivity Disorder (ADHD). Front. Hum. Neurosci..

[7] Tulving E., Craik F.I.M. (2000). The Oxford Handbook of Memory.

[8] Hervey A.S., Epstein J.N., Curry J.F. (2004). Neuropsychology of Adults With Attention-Deficit/Hyperactivity Disorder: A Meta-Analytic Review. Neuropsychology.

[9] Schoechlin C., Engel R.R. (2005). Neuropsychological Performance in Adult Attention-Deficit Hyperactivity Disorder: Meta-Analysis of Empirical Data. Arch. Clin. Neuropsychol..

[10] Andersen P.N., Hovik K.T., Skogli E.W., Egeland J., Øie M. (2013). Symptoms of ADHD in Children with High-Functioning Autism Are Related to Impaired Verbal Working Memory and Verbal Delayed Recall. PLoS ONE.

[11] Cutting L.E., Koth C.W., Mahone E.M., Denckla M.B. (2003). Evidence for Unexpected Weaknesses in Learning in Children with Attention-Deficit/Hyperactivity Disorder without Reading Disabilities. J. Learn. Disabil..

[12] Kibby M.Y., Cohen M.J. (2008). Memory Functioning in Children with Reading Disabilities and/or Attention-Deficit/Hyperactivity Disorder: A Clinical Investigation of Their Working Memory and Long-Term Memory Functioning. Child Neuropsychol..

[13] Kofler M.J., Singh L.J., Soto E.F., Chan E.S.M., Miller C.E., Harmon S.L., Spiegel J.A. (2020). Working Memory and Short-Term Memory Deficits in ADHD: A Bifactor Modeling Approach. Neuropsychology.

[14] Lee S.E., Kibby M.Y., Cohen M.J., Stanford L., Park Y., Strickland S. (2016). Differences in Memory Functioning between Children with Attention-Deficit/Hyperactivity Disorder and/or Focal Epilepsy. Child Neuropsychol..

[15] Zarghi A., Mehrinejad S.A., Zali A., Ramezankhani Z. (2012). Memory Performance among Children with ADHD. Basic Clin. Neurosci..

[16] Jansen P., Wiedenbauer G., Hahn N. (2010). Manual Rotation Training Improves Direction-Estimations in a Virtual Environmental Space. Eur. J. Cogn. Psychol..

[17] Alpanda S. (2015). The Investigation of the Relationship between ADHD and Visual-Spatial Functions. Procedia-Soc. Behav. Sci..

[18] Cardillo R., Vio C., Mammarella I.C. (2020). A Comparison of Local-Global Visuospatial Processing in Autism Spectrum Disorder, Nonverbal Learning Disability, ADHD and Typical Development. Res. Dev. Disabil..

[19] Cundari M., Vestberg S., Hansson A., Kennberg J., Gustafsson P., Rasmussen A. (2025). Sensorimotor Functions, Visuospatial Perception and Visuospatial Abilities in Adult Attention Deficit Hyperactivity Disorder and Autism Spectrum Disorder. J. Int. Neuropsychol. Soc..

[20] Semrud-Clikeman M., Walkowiak J., Wilkinson A., Christopher G. (2010). Neuropsychological Differences among Children with Asperger Syndrome, Nonverbal Learning Disabilities, Attention Deficit Disorder, and Controls. Dev. Neuropsychol..

[21] Blankenship T.L., Calkins S.D., Bell M.A. (2022). The Role of Executive Functions in Item Recognition and Temporal Order Memory Development. J. Cogn. Dev. Off. J. Cogn. Dev. Soc..

[22] Garcia N., Dick A.S., Pruden S.M. (2022). Contributions of Executive Function to Spatial Thinking in Young Children. Infant Child Dev..

[23] Becker S.P., Willcutt E.G., Leopold D.R., Fredrick J.W., Smith Z.R., Jacobson L.A., Burns G.L., Mayes S.D., Waschbusch D.A., Froehlich T.E. (2023). Report of a Work Group on Sluggish Cognitive Tempo: Key Research Directions and a Consensus Change in Terminology to Cognitive Disengagement Syndrome. J. Am. Acad. Child Adolesc. Psychiatry.

[24] Cerny B.M., Reynolds T.P., Chang F., Scimeca L.M., Phillips M.S., Ogram Buckley C.M., Leib S.I., Resch Z.J., Pliskin N.H., Soble J.R. (2023). Cognitive Performance and Psychiatric Self-Reports Across Adult Cognitive Disengagement Syndrome and ADHD Diagnostic Groups. J. Atten. Disord..

[25] Croghan A. (2022). Sluggish Cognitive Tempo in a Pediatric Population. Doctoral Dissertation.

[26] Tamm L., Brenner S.B., Bamberger M.E., Becker S.P. (2018). Are Sluggish Cognitive Tempo Symptoms Associated with Executive Functioning in Preschoolers?. Child Neuropsychol..

[27] Read N., Mulraney M., McGillivray J., Sciberras E. (2020). Comorbid Anxiety and Irritability Symptoms and Their Association with Cognitive Functioning in Children with ADHD. J. Abnorm. Child Psychol..

[28] Panvino F., Zaccaria V., Pica M., Amitrano N., Pisani F., Di Brina C. (2025). ADHD Children Take More Time to Inhibit Automatic Responses: A Comparison with Anxiety Disorders Using NEPSY-II. Children.

[29] Kaufman J., Birmaher B., Brent D., Rao U., Flynn C., Moreci P., Williamson D., Ryan N. (1997). Schedule for Affective Disorders and Schizophrenia for School-Age Children—Present and Lifetime Version (K-SADS-PL): Initial Reliability and Validity Data. J. Am. Acad. Child Adolesc. Psychiatry.

[30] Ünal F., Öktem F., Çetin Çuhadaroğlu F., Çengel Kültür S.E., Akdemir D., Foto Özdemir D., Çak H.T., Ünal D., Tıraş K., Aslan C. (2019). Reliability and Validity of the Schedule for Affective Disorders and Schizophrenia for School-Age Children-Present and Lifetime Version, DSM-5 November 2016-Turkish Adaptation (K-SADS-PL-DSM-5-T). Turk. J. Psychiatry.

[31] Turgay A. (1994). Disruptive Behavior Disorders Child and Adolescent Screening and Rating Scales for Children, Adolescents, Parents and Teachers.

[32] Ercan E.S., Amado S., Somer O., Cıkoğlu S. (2001). Development of A Test Battery for the Assessment of Attention Deficit Hyperactivity Disorder. Turk. J. Child Adolesc. Psychol..

[33] Barkley R.A. (2013). Distinguishing Sluggish Cognitive Tempo from ADHD in Children and Adolescents: Executive Functioning, Impairment, and Comorbidity. J. Clin. Child Adolesc. Psychol..

[34] Firat S., Bolat G.U., Gul H., Baytunca M.B., Kardas B., Aysev A., Ercan E.S. (2018). Barkley Child Attention Scale Validity and Reliability Study. Dusunen Adam J. Psychiatry Neurol. Sci..

[35] Birmaher B., Khetarpal S., Brent D., Cully M., Balach L., Kaufman J., Neer S.M. (1997). The Screen for Child Anxiety Related Emotional Disorders (SCARED): Scale Construction and Psychometric Characteristics. J. Am. Acad. Child Adolesc. Psychiatry.

[36] Karaceylan F. (2005). Reliability and Validity of SCARED in Turkish Children. Ph.D. Thesis.

[37] Öktem Ö. (2011). Öktem Verbal Memory Processes Test (ÖKTEM-VMPT) Manual.

[38] Usta A.Ö. (2016). Collecting Normative Data of the Verbal Memory Processes Test (SBST) from Children Aged 6–9 Years. Master’s Thesis.

[39] Wechsler D. (1997). Wechsler Memory Scale—Third Edition (WMS–III): Administration and Scoring Manual.

[40] Strauss E., Sherman E.M.S., Spreen O. (2006). A Compendium of Neuropsychological Tests: Administration, Norms, and Commentary.

[41] Wechsler D. (2003). Wechsler Intelligence Scale for Children—Fourth Edition (WISC-IV).

[42] Martin J., Langley K., Cooper M., Rouquette O.Y., John A., Sayal K., Ford T., Thapar A. (2024). Sex Differences in Attention-Deficit Hyperactivity Disorder Diagnosis and Clinical Care: A National Study of Population Healthcare Records in Wales. J. Child Psychol. Psychiatry.

[43] Krone B., Adler L.A., Anbarasan D., Leon T., Gallagher R., Patel P., Faraone S.V., Newcorn J.H. (2023). Characteristics of Sluggish Cognitive Tempo among Adults with ADHD: Objective Neurocognitive Measures Align with Self-Report of Executive Function. Front. Child Adolesc. Psychiatry.

[44] Burns G.L., Servera M., Bernad M.D.M., Carrillo J.M., Cardo E. (2013). Distinctions between Sluggish Cognitive Tempo, ADHD-IN, and Depression Symptom Dimensions in Spanish First-Grade Children. J. Clin. Child Adolesc. Psychol..

[45] Baytunca M.B., Inci S.B., Ipci M., Kardas B., Bolat G.U., Ercan E.S. (2018). The Neurocognitive Nature of Children with ADHD Comorbid Sluggish Cognitive Tempo: Might SCT Be a Disorder of Vigilance?. Psychiatry Res..

[46] Uytun M.C., Yurumez E., Babayigit T.M., Efendi G.Y., Kilic B.G., Oztop D.B. (2023). Sluggish Cognitive Tempo Symptoms Cooccurring with Attention Deficit Hyperactivity Disorder. Middle East Curr. Psychiatry.

[47] Skirbekk B., Hansen B.H., Oerbeck B., Kristensen H. (2011). The Relationship between Sluggish Cognitive Tempo, Subtypes of Attention-Deficit/Hyperactivity Disorder, and Anxiety Disorders. J. Abnorm. Child Psychol..

[48] Wu Z.-M., Liu J., Wang P., Wang Y.-F., Yang B.-R. (2022). Neuropsychological Characteristics of Children with Attention-Deficit/Hyperactivity Disorder and Sluggish Cognitive Tempo. J. Atten. Disord..

[49] Inci Izmir S.B., Aktan Z.D., Ercan E.S. (2024). The Comparison of Psychological Factors and Executive Functions of Children with Attention Deficit Hyperactivity Disorder and Cognitive Disengagement Syndrome to ADHD and ADHD Comorbid with Oppositional Defiant Disorder. J. Atten. Disord..

[50] Yazan Songür Ç. (2022). Evaluation of the Neuropsychological Profiles of Patients Diagnosed with Sluggish Cognitive Tempo. Unpublished Medical Specialty Thesis.

[51] Ünsel Bolat G., Ercan E.S., Bolat H., Süren S., Bacanlı A., Yazici K., Rohde L. (2019). Comparisons between Sluggish Cognitive Tempo and ADHD-Restrictive Inattentive Presentation Phenotypes in a Clinical ADHD Sample. ADHD Atten. Deficit Hyperact. Disord..

[52] Becker S.P., Leopold D.R., Burns G.L., Jarrett M.A., Langberg J.M., Marshall S.A., McBurnett K., Waschbusch D.A., Willcutt E.G. (2016). The Internal, External, and Diagnostic Validity of Sluggish Cognitive Tempo: A Meta-Analysis and Critical Review. J. Am. Acad. Child Adolesc. Psychiatry.

[53] Weiss L.G., Schwartz D.M., Prifitera A., Courville T., Saklofske D.H., Weiss L.G., Prifitera A., Holdnack J.A., Saklofske D.H. (2006). 4-Advanced Clinical Interpretation of WISC-IV Index Scores. WISC-IV Advanced Clinical Interpretation.

[54] Anning K.L., Langley K., Hobson C., De Sonneville L., Van Goozen S.H.M. (2024). Inattention Symptom Severity and Cognitive Processes in Children at Risk of ADHD: The Moderating Role of Separation Anxiety. Child Neuropsychol..

[55] Gau S.S.-F., Chiang H.-L. (2013). Association between Early Attention-Deficit/Hyperactivity Symptoms and Current Verbal and Visuo-Spatial Short-Term Memory. Res. Dev. Disabil..

[56] Pawley A.D., Mayer J.S., Medda J., Brandt G.A., Agnew-Blais J.C., Asherson P., Rommel A.-S., Ramos-Quiroga J.A., Palacio Sanchez J., Bergsma D. (2024). Verbal Memory Performance in Adolescents and Adults with ADHD. Neurosci. Appl..

[57] Semrud-Clikeman M. (2012). The Role of Inattention on Academics, Fluid Reasoning, and Visual–Spatial Functioning in Two Subtypes of ADHD. Appl. Neuropsychol. Child.

[58] Eysenck M.W., Calvo M.G. (1992). Anxiety and Performance: The Processing Efficiency Theory. Cogn. Emot..

[59] Eysenck M.W., Derakshan N., Santos R., Calvo M.G. (2007). Anxiety and Cognitive Performance: Attentional Control Theory. Emotion.

