# Supplementary material for: Neurocognitive Correlates of Diagnostic Heterogeneity in Children with ADHD: The Differential Contributions of Cognitive Disengagement Syndrome, Symptom Severity, and Anxiety
Source: Diagnostics (Basel). 2026 Mar 9;16(5):808. doi: 10.3390/diagnostics16050808 (PMC12984921; doi:10.3390/diagnostics16050808)
Supplement: Supplementary file 1 [file diagnostics-16-00808-s001.zip › diagnostics-4087776-supplement.pdf]

Table S1 presents the correlations between ADHD symptom scores (derived from combined parent and teacher T-DSM-IV-S ratings), anxiety scores (SCARED–total), and neuropsychological test performances, in the total sample. Inattention scores showed moderate, negative, and significant correlations with WMS-VR STM and JLO-Total Score (r = -.307 and r = -.360, respectively; $p < .001$), as well as negative, low-level, significant correlations with OVMPT-Immediate Memory (r = -.257, $p < .01$), OVMPT-Total Learning (r = -.203, $p < .05$), OVMPT-Spontaneous Recall (r = -.261, $p < .01$), and WISC-IV Block Design (r = -.236, $p < .05$). In addition, a low-level, positive and significant correlation was observed between Inattention scores and OVMPT-False Learning (r = .216, $p < .05$). No significant correlations were found between the hyperactivity/impulsivity scores and any of the neuropsychological test measures ($p > .05$). Regarding anxiety, a moderate, positive, and significant correlation was found with OVMPT-Total Learning (r = 0.311, $p < .001$), while low-level, positive and significant correlations were observed with OVMPT-Highest Learning (r = .286, $p < .01$), OVMPT-Spontaneous Recall (r = .229, $p < .05$), and WMS-VR STM (r = .203, $p < .05$). No other significant associations were detected between anxiety scores and the remaining neuropsychological measures ($p > .05$).

Table S1. *Correlations Between ADHD Symptom Severity, Anxiety Severity, and Neuropsychological Test Performances (n=120)*

Neuropsychological Tests	Inattention Symptom Severity	Hyperactivity/Impulsivity Symptom Severity	Anxiety Symptom Severity
OVMPT- Immediate Memory[a]	**-.257****	-.016	.162
OVMPT- Total Learning[b]	**-.203***	-.029	**.311*****
OVMPT-Highest Learning[a]	-.167	-.032	**.286****
OVMPT- False Learning[b]	**.216***	.016	-.063
OVMPT-Perseveration[b]	.037	.005	-.169
OVMPT- Spontaneous Recall[b]	**-.261****	-.115	**.229***
OVMPT- False Recall[b]	.145	-.052	-.119
OVMPT- Total Recall[b]	.023	.097	.016
OVMPT- False Recognition[b]	.054	.015	.013
WMS–VR STM[a]	**-.307*****	-.132	**.203***
WISC-IV Block Design[a]	**-.236***	.070	-.013
JLO-Total Score[a]	**-.360*****	-.173	-.011

Note. OVMPT = Oktem Verbal Memory Processes Test; WMS–VR STM = Wechsler Memory Scale–Visual Reproduction Short-Term Memory; WISC–IV Block Design = Wechsler Intelligence Scale for Children–Fourth Edition, Block Design; JLO = Judgment of Line Orientation. Symptom severity scores for Inattention and Hyperactivity/Impulsivity were derived from the T-DSM-IV-S (combined parent–teacher ratings), and anxiety symptom severity scores were obtained from the total score of the SCARED. *a* = Pearson correlation; *b* = Spearman correlation. Correlation coefficients (*r*) are presented in the table. Bold values indicate significant correlations (**p* <.05; *****p*** <.01; ****p* < .001).
